# The dilemma of sweet temptation: How sugar perception confusion in sweetened beverages shapes consumer avoidance behavior

**DOI:** 10.1371/journal.pone.0336953

**Published:** 2025-12-16

**Authors:** Qingxia Li, Xin Yang, Xin Bai, Ying Zhang, Qiang Li, Yingji Li

**Affiliations:** 1 School of Government Management, Henan University of Economics and Law, Zhengzhou, China; 2 Graduate School, Stamford International University, Bangkok, Thailand; 3 College of Architectural Arts, Guangxi Arts University, Nanning, China; 4 Department of Public Education, Shandong Vocational College of Special Education, Jinan, China; 5 School of Economics and Management, Shanghai Technical Institute of Electronics & Information, Shanghai, China; 6 School of Humanities and Management, Yunnan University of Chinese Medicine, Kunming, China; Lusofona University of Humanities and Technologies: Universidade Lusofona de Humanidades e Tecnologias, PORTUGAL

## Abstract

Despite widespread consumption of sugar-sweetened beverages, consumers face contradictory information from health authorities, marketing, and social media, yet limited research examines how this information conflict affects purchasing decisions. This study investigates how sugar perception confusion influences purchasing avoidance through ambivalent attitudes. Based on cognitive dissonance and information processing theories, we developed a cognitive-affective-behavioral model examining relationships among sugar perception confusion, ambivalent attitudes, and purchasing avoidance behaviors. Using PLS-SEM analysis of 531 Chinese consumers, results show sugar perception confusion significantly affects ambivalent attitudes (β = 0.576, p < 0.001), which strongly predict purchasing avoidance (β = 0.593, p < 0.001). Sugar perception confusion also directly influences purchasing avoidance (β = 0.155, p < 0.001), with ambivalent attitudes serving as a significant mediator (indirect effect β = 0.342, p < 0.001). These findings advance consumer information processing theory and provide evidence-based insights for optimizing information environments to support informed decision-making.

## 1. Introduction

Modern consumers navigate increasingly complex information environments where conflicting messages from multiple sources challenge effective decision-making. This complexity is particularly evident in sugar-sweetened beverage consumption, where health warnings, marketing claims, and social media content often contradict each other. Despite improved information accessibility, global consumption continues rising, especially among educated demographics [1], suggesting that information abundance may paradoxically impair rather than improve decision quality. This information conflict problem is particularly prominent in real consumer environments. Sugar-sweetened beverage labeling exemplifies this complexity through inconsistent measurement units, diverse sugar terminology, and contradictory nutritional claims that contrast sharply with public health agencies’ simplified health warnings, creating a typical information conflict environment. This discrepancy between complex product information and simplified health messaging generates cognitive confusion that fundamentally disrupts consumers’ ability to make informed decisions, leading to emotional conflicts and ultimately influencing purchasing behaviors.

In the modern information ecosystem, consumers are simultaneously exposed to conflicting sugar-related information: health departments emphasize sugar intake risks, the food industry promotes sugar’s functional benefits, and social media disseminates unverified health claims. Surveys show that while 73% of consumers report understanding sugar intake health risks, 63% still consume sugar-sweetened beverages daily [2]. This knowledge-behavior dissociation indicates that the core problem lies not in information scarcity, but in how complex information environments impair consumers’ decision-making capabilities themselves.

Sugar-sweetened beverages, defined as beverages containing added sugars including sodas, fruit drinks, sweetened teas, and energy drinks [3], represent an ideal context for studying information conflict effects due to their widespread consumption and the intense public health attention they receive. The consumption patterns of these beverages have increased globally across all age groups between 2016 and 2023, with young consumers being particularly affected by social media influences on food choices [1,4]. This creates a natural laboratory for examining how consumers process conflicting information about products they enjoy but increasingly recognize as potentially harmful.

However, current consumer information processing research has significant gaps in understanding consumers’ psychological response mechanisms in information conflict environments, limiting the ability to improve information environment design. First, existing research lacks systematic measurement of domain-specific confusion phenomena in complex information environments, particularly sugar perception confusion—the cognitive confusion state when processing conflicting sugar-related health information. Second, the psychological transmission mechanism between sugar perception confusion and consumers’ behavioral responses lacks systematic explanation, particularly how ambivalent attitudes serve as key mediating variables. Third, existing research lacks systematic analysis of the complete psychological pathway from information conflict exposure to behavioral responses, particularly insufficient understanding of how cognitive confusion in complex information environments leads to specific avoidance behaviors through emotional mediating processes [5].

These theoretical gaps constrain consumer information processing theory development and hinder information environment designers and policymakers from optimizing information disclosure strategies based on evidence-based research. Understanding and improving information environments’ decision support effects has become an urgent scientific issue for enhancing consumer welfare.

To address these gaps and advance our understanding of consumer psychology in information conflict environments, this research aims to systematically analyze the psychological mechanisms among sugar perception confusion, ambivalent attitudes, and purchasing avoidance behaviors in complex information environments, Sugar perception confusion refers to the cognitive confusion state consumers experience when processing conflicting sugar-related information. Ambivalent attitudes represent the simultaneous positive and negative evaluations consumers hold toward sugar-sweetened beverages. Purchasing avoidance behavior encompasses both delaying and abandoning purchase decisions due to decision-making difficulties. Specifically, this study examines how sugar perception confusion directly affects consumers’ ambivalent attitudes (H1), how ambivalent attitudes lead to purchasing avoidance behaviors (H2), and the direct effects of sugar perception confusion on purchasing avoidance behaviors (H3).

This study’s theoretical contributions include: first, extending cognitive dissonance theory to complex information environments by demonstrating how information conflicts generate specific psychological response patterns; second, developing a domain-specific measurement framework for information confusion that can be applied to other health-related product categories; third, validating a complete mediation model linking information environment quality to consumer behavior through emotional pathways. The practical value lies in providing evidence-based guidelines for information environment design that support rather than hinder consumer decision-making, offering policymakers concrete strategies for information disclosure that enhance consumer welfare, and contributing actionable frameworks for optimizing digital age information environments to support informed health decisions.

## 2. Literature review and theoretical hypothesis

### 2.1. Literature review

#### 2.1.1. Sugar perception confusion in complex information environments.

Consumer information processing research indicates that when consumers are simultaneously exposed to diversified and conflicting information, they develop specific cognitive confusion states [6]. This phenomenon is particularly prominent in sugar-related information communication. In recent years, consumers have simultaneously received sugar-related information from multiple sources: public health agencies emphasize the health risks of sugar intake [7], the food industry promotes the functional benefits of sugar, and social media disseminates various unverified health claims. This information conflict environment has become an important factor affecting consumer cognition.

Consumer confusion, as a manifestation of information processing barriers, originally originated from psychological research [8] and later developed from the information overload paradigm in marketing [9,10]. However, confusion phenomena in complex information environments have unique characteristics that differ from general consumer confusion [11]. Sugar perception confusion, as domain-specific confusion in information environments, specifically refers to the cognitive confusion state that consumers experience when processing conflicting information about sugar, directly reflecting the quality problems of current information environments.

Cognitive dissonance theory [12] provides a theoretical foundation for understanding this phenomenon. The theory indicates that when individuals simultaneously receive conflicting information, psychological tension arises, driving them to seek ways to reduce inconsistency. In complex information environments, this psychological tension manifests as perceived confusion about information, which is a direct cognitive manifestation of information conflict. Consumer confusion is characterized as a state of mental disarray stemming from external market stimuli such as technological complexity, leading to a perplexing decision-making situation [13].

With the global increase in awareness of health risks associated with sugar-sweetened beverages, related information communication activities have significantly increased. The United States conducted mass media campaigns in rural areas to emphasize the dangers of sugar-sweetened beverages [14], Australia conducted public awareness activities highlighting the detrimental effects of sugar-sweetened beverages on health [15], and the United Kingdom published multiple articles discussing the harmful effects of sugar-sweetened beverages on health [16]. However, these information communication efforts conflict with consumers’ daily experiences and commercial information, leading consumers to develop cognitive confusion about sugar-related information. This study defines sugar perception confusion as: the cognitive confusion and psychological uncertainty state that consumers experience when exposed to conflicting sugar-related information, serving as an important cognitive indicator reflecting the quality problems of information environments.

#### 2.1.2. Mechanism of ambivalence attitude formation in information conflict environments.

The concept of ambivalence attitude was initially introduced into social psychology by Scott [17], and research on consumer attitudes began in 1997 [18]. Ambivalence attitude is defined as a complex emotional state that consumers develop instantaneously or gradually, reflecting the two-dimensional nature of consumer attitudes, including positive and negative evaluations of the same product. Scholars believe that ambivalence attitudes arise when decision-makers simultaneously evaluate a problem positively and negatively [19].

In complex information environments, the formation mechanism of ambivalence attitudes can be deeply explained through cognitive dissonance theory. When consumers face conflicts between hedonic needs and health goals, they simultaneously hold positive and negative attitude evaluations [20]. This conflict is not a personal defect of consumers but an inevitable product of complex information environments. In sugar-sweetened beverage consumption, ambivalence attitudes reflect the conflict effects between multi-source information (such as sensory experiences, commercial marketing, health information and social identity).

Consumer information processing research has found that inconsistent information leads consumers to develop complex emotional states, recognizing health risks while maintaining consumption preferences [21]. The contradictory nature of sugar makes this emotional conflict more prominent: sugar is both perceived as pleasurable and necessary and also viewed as addictive and harmful [21]. Multiple studies have focused on the ambivalence attitudes arising from sugar-sweetened beverages and their health implications, including ambivalence attitudes toward sugar-sweetened beverages among students in the context of information interventions [22], parents’ ambivalence attitudes toward sugar-sweetened beverages [23], and ambivalence attitudes toward sugar-sweetened beverages among African American and Latino adolescents in the United States [24].

From an information environment design perspective, ambivalence attitudes not only reflect consumers’ inner emotional conflicts but more importantly reflect the impact mechanisms and quality level of current information environments at the emotional level. Ideal information environments should help consumers resolve rather than exacerbate emotional conflicts, supporting them in making autonomous choices based on clear cognition. Based on the definitions of De Liver et al., this study defines ambivalence attitude as: the coexistence of positive and negative cognitive evaluations and emotional experiences of an individual towards the same attitude object [25]. In complex information environments, this specifically refers to consumers’ simultaneous positive and negative attitudes toward sugar-sweetened beverages, reflecting the conflict effects between multi-source information.

#### 2.1.3. Behavioral indicators of information environment quality: purchasing avoidance behavior.

The concept of purchasing avoidance originated from Beattie et al.‘s 1994 definition of decision-making attitude, which refers to the desire to make or avoid a decision regardless of the outcome achieved by the decision [26]. In subsequent studies, purchasing avoidance was further defined as the behavior of not purchasing any available options, including not purchasing at all (i.e., abandonment) or purchasing later (i.e., postponement) [27–29].

When consumers search for information, analyze the acquired information, and process product information, negative evaluations of planned purchase decisions may lead to delays or cancellations in the decision-making process [30]. Taking the selection and purchase of sugar-sweetened beverages as an example, although consumers enjoy the taste of sugar, the external information they now receive may lead them to conclude that sugar can harm their physical health.

In complex information environments, the formation mechanism of purchasing avoidance behavior has special significance. Information processing theory indicates that when consumers face conflicting or ambiguous information, it increases the cognitive burden of information processing, leading to negative evaluations of purchase decisions [31]. These negative evaluations do not necessarily reflect consumers’ true preferences for the products themselves but may stem from the complexity and uncertainty of the information environment.

This study defines purchasing avoidance as: the delay, cancellation, or perceived stress in the decision-making process resulting from negative evaluations of planned purchase decisions after consumers search, analyze, and process product information. This definition includes two core dimensions: delaying purchase (postponing purchase decisions) and abandoning purchase (canceling purchase decisions), while encompassing the psychological stress that consumers experience during this process.

From an information environment quality assessment perspective, purchasing avoidance behavior is an important indicator of consumers’ information processing difficulties. When information environment design leads consumers to experience stress, delays, or avoidance in the decision-making process, this suggests that information may have conflicts, ambiguity, or complexity issues, requiring further optimization of information environment design to better support consumer decision-making.

### 2.2. Theoretical hypotheses

#### 2.2.1. The relationship between sugar perception confusion and ambivalence attitude.

Cognitive dissonance theory [12] predicts that when individuals simultaneously receive conflicting information, psychological tension arises, which then triggers conflict responses at the emotional level. In complex information environments, sugar perception confusion, as a cognitive manifestation of information conflict, directly leads to the formation of ambivalence attitudes at the emotional level. This mechanism reflects how conflicts between multi-source information and consumers’ existing cognition and preferences generate complex psychological impacts. Consumer information processing research demonstrates that conflicting information triggers complex emotional responses in consumers [32]. When consumers feel confused about sugar-related information, they develop complex emotions toward sugar-sweetened beverages—both liking and worrying: on one hand enjoying the pleasurable experience that sugar brings, on the other hand worrying about health risks. This is a typical psychological mechanism of cognitive confusion transforming into emotional conflict.

The contradictory nature of sugar exacerbates this psychological conflict. Because sugar is both perceived as pleasurable and necessary and also viewed as addictive and harmful [21], when consumers receive conflicting information about sugar, they are more likely to develop cognitive confusion, which then triggers ambivalence attitudes toward sugar-sweetened beverages. These attitudes reflect the complex psychological state of consumers simultaneously recognizing both positive attributes of sugar (taste pleasure, energy supply) and negative attributes (health risks, addiction).

From a consumer information processing perspective, this transformation process from cognitive confusion to ambivalence attitudes reveals an important problem with complex information environments: excessive conflicting information not only fails to help consumers form clear cognition but actually create more psychological conflicts at the emotional level. Understanding this mechanism is of great significance for redesigning information environments.

Therefore, this study proposes the following hypothesis:

H1: Sugar perception confusion has a significant positive effect on ambivalence attitude.

#### 2.2.2. The relationship between ambivalence attitude and purchasing avoidance behavior.

Emotional conflict theory indicates that when individuals hold ambivalent attitudes toward the same object, they experience psychological discomfort and decision-making difficulties, driving them to adopt specific behavioral strategies to reduce this discomfort [33]. Consumer decision-making research has found that emotional conflicts when facing complex choices lead to behavioral avoidance and decision delays [20].

Empirical research supports the causal relationship between ambivalent attitudes and avoidance behaviors. Voting studies have found that individuals experiencing ambivalence are more likely to tend towards procrastination [34]. Consumer research shows that making choices when holding ambivalent attitudes leads to psychological discomfort [35]. When consumers’ attitudes are strongly ambivalent, they have significant motivation to reduce this ambivalence and may adopt strategies such as delaying or avoidance [33].

In complex information environments, when consumers hold ambivalent attitudes toward sugar-sweetened beverages, they experience cognitive-emotional conflicts. These ambivalent attitudes lead consumers to develop negative evaluations of purchase decisions, which then manifest as purchasing avoidance behaviors. Specifically, ambivalent attitudes increase the complexity and psychological burden of decision-making, making consumers tend to delay purchase decisions (avoiding current psychological conflicts through postponement) or abandon purchase decisions (reducing decision pressure through withdrawal).

From an information environment quality assessment perspective, purchasing avoidance caused by ambivalent attitudes reflects the limitations of information environments in supporting consumer decision-making. When consumers experience decision pressure and avoidance behaviors due to ambivalent attitudes, this indicates that current information environments may have conflicts or complexity and fail to effectively integrate consumers’ cognitive and emotional needs.

Therefore, this study proposes the following hypothesis

H2: Ambivalence attitude has a significant positive effect on purchasing avoidance behavior.

#### 2.2.3. The relationship between sugar perception confusion and purchasing avoidance behavior.

Information processing theory [10] indicates that cognitive confusion itself is sufficient to damage the quality and efficiency of decision-making processes, even without considering the mediating role of emotional factors. In complex information environments, sugar perception confusion, as an information processing barrier, directly leads to decision difficulties and behavioral avoidance. This direct effect reflects the fundamental impact of information environment quality on consumers’ decision-making capabilities.

Consumers who may experience or are prone to confusion may consciously or unconsciously adopt various methods to reduce confusion in order to prevent potential adverse outcomes [31]. When consumers feel confused during the decision-making process, they are less likely to make rational purchase decisions and may encounter adverse outcomes such as delaying/abandoning purchases, buying wrong products, experiencing cognitive dissonance or shopping fatigue [11,31].

In complex information environments, this mechanism has special significance. When consumers face conflicting information about sugar, the processing capacity of cognitive systems is challenged, leading to delays or interruptions in the decision-making process. This phenomenon differs from rational decision-making based on sufficient information and is a direct behavioral manifestation of information environment quality problems. Sugar perception confusion directly affects information processing efficiency, leading consumers to choose to delay purchases (trying to obtain more clear information) or abandon purchases (withdrawing from complex decision-making processes).

Consumer information processing research confirms that information conflict environments directly affect consumers’ behavioral choices, leading to decision delays or abandonment [31]. The existence of this direct effect emphasizes the urgency of optimizing information environment quality and the importance of reducing the damage that information conflicts inflict on consumers’ decision-making capabilities. From an information environment design perspective, understanding this direct mechanism helps identify key intervention points for improving information environment quality. Therefore, this study proposes the following hypothesis:

H3: Sugar perception confusion has a significant positive effect on purchasing avoidance behavior.

**Fig 1:**
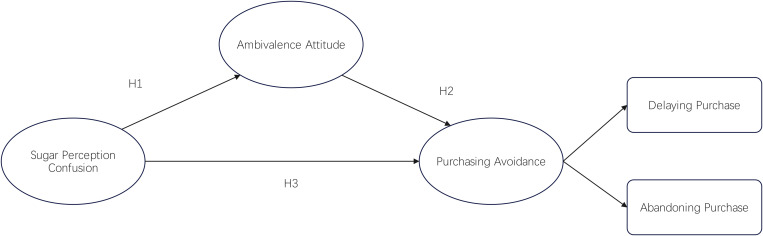
Research model. Note: H1–H3 represent hypothesized paths. H1: the effect of sugar perception confusion on ambivalence attitude; H2: the effect of ambivalence attitude on purchasing avoidance; H3: the direct effect of sugar perception confusion on purchasing avoidance.

Fig 1 illustrates the research model of this study, which focuses on exploring the relationships between sugar perception confusion, ambivalence attitude, and purchasing avoidance.

## 3. Methodology

### 3.1. Participants and procedure

The study conducted its research online, utilizing the Wenjuanxing website (https://www.wjx.cn/) to distribute survey questionnaires. This research employed purposive (judgmental) sampling to ensure participants met specific research requirements. An online survey was distributed through multiple channels, including an online survey platform (Wenjuanxing), social media platforms (WeChat and Weibo), and university and community networks in target cities. Inclusion criteria required participants to be adults aged 18 and above, residents of China’s first-tier cities (Beijing, Shanghai, Guangzhou), consumers who had purchased or consumed sugar-sweetened beverages within the past three months, and capable of independently completing online surveys. Exclusion criteria eliminated individuals under 18 years old, non-first-tier city residents, those without recent sugar-sweetened beverage consumption experience, and those unable to understand survey content. Quality control mechanisms included IP address detection to avoid duplicates, minimum completion time requirements (120 seconds), logical consistency checks, and manual review of open-ended questions. Over three months, from August to November 2023, the survey targeted individuals who had consumed sugar-sweetened beverages in major first-tier cities in China. A total of 647 online questionnaires were distributed, resulting in 531 valid responses with an effective response rate of 82.1% after eliminating invalid responses. The collected sample met the criteria for sample size established [36], ensuring its adequacy for statistical analysis. Furthermore, the large sample size significantly enhanced the statistical power and robustness of the analysis [37].

The survey questionnaire comprised two sections. Section 1 focused on gathering demographic information from the participants, while Section 2 delved into detailed questions designed to measure variables within the proposed research model. The measurement items for these constructs were adopted from well-established scales developed by Ermeç Sertoğlu and Kavak [38]; Priester and Petty [39]; Walsh and Mitchell [11]. The survey employed a Likert scale ranging from 1 (strongly disagree) to 7 (strongly agree) to assess the various constructs within the study.

### 3.2. Measures and data analysis

The questionnaire items in the study are presented in Table 1. This study utilizes Partial Least Squares-Structural Equation Modeling (PLS-SEM) for data analysis. This research chose PLS-SEM over CB-SEM based on four considerations: (1) theory-based prediction focus – this study aims to predict variance in behavioral outcomes based on established theory, making PLS-SEM’s prediction-oriented approach more suitable [40]; (2) sample characteristics – PLS-SEM can efficiently handle the moderate sample size (n = 531) and achieve robust results; (3) measurement model – all constructs employ reflective indicators, aligning well with PLS-SEM’s component-based approach; (4) research objective – the focus is on predicting variance in endogenous constructs rather than achieving perfect model fit, making PLS-SEM a suitable choice [40]. PLS-SEM is an iterative estimation method that combines principal component analysis with multiple regression, making it a powerful tool for causal modeling [37]. One of the advantages of PLS is its relatively lenient requirements for variable normality and randomness, along with its ability to analyze complex predictive models [40–43].

**Table 1:** Questionnaire items and references.

Variables	code	Measurement Questions	References sources
Sugar Perception Confusion	SPC1	Drinking sugar-sweetened beverages confuses me.	Ermeç Sertoğlu and Kavak [38]
	SPC2	Drinking sugar-sweetened beverages brings joy, but I am concerned about the presence of sugar.	
	SPC3	Drinking sugar-sweetened beverages is enjoyable, but I am worried that excessive sugar intake may pose health risks.	
Ambivalence Attitude	AA1	Because of the presence of sugar, I hesitate to buy sugar-sweetened beverages.	Priester and Petty [39]
	AA2	I feel conflicted about whether to buy sugar-sweetened beverages due to the presence of sugar.	
	AA3	I have both positive and negative opinions about sugar-sweetened beverages because of the presence of sugar.	
Delaying Purchase	DP1	Buying sugar-sweetened beverages is difficult because of the sugar information.	Walsh and Mitchell [11]
	DP2	I have to think twice before buying sugar-sweetened beverages because of the sugar information.	
	DP3	Sugar information make the purchasing decision taking a few seconds longer than expected.	
Abandoning Purchase	AP1	Due to sugar information, I no longer pay attention to sugar-sweetened beverages.	
	AP2	I choose to buy other beverages because of sugar information.	
	AP3	I refuse to buy sugar-sweetened beverages because of sugar information.	

This study conducts the PLS-SEM analysis and estimation process in two distinct phases. The first phase focuses on assessing the questionnaire’s reliability and validity, ensuring the collected data’s quality and accuracy. The second phase involves calculating and validating the structural model’s path coefficients and explanatory power. These two phases are designed to validate the structural relationships proposed in the study [44–45]. Specifically, this research explores the relationships between sugar perception confusion, ambivalence attitudes, and purchasing avoidance.

## 4. Results

### 4.1 Descriptive analysis and non-response bias

#### 4.1.1. Descriptive analysis.

Among the 531 valid questionnaires, 326 (61.4%) were female respondents and 205 (38.6%) were male respondents. The age group was between 18 and 25 (340 people, accounting for 64.03%). The monthly disposable income was mainly below 3,000 yuan (306 people, accounting for 57.6%). The education level was mainly concentrated at the undergraduate level (336 people, accounting for 63.28%). (See Table 2)

**Table 2:** Demographics.

Sample	Category	Frequency	Percentage
Gender	Male	205	38.6
	Female	326	61.4
Age	Under 18 years old	74	13.9
	18 ~ 25	340	64.0
	26 ~ 30	62	11.7
	31 ~ 40	31	5.8
	41 ~ 50	12	2.3
	Over 50	12	2.2
Monthly disposable income	3000 and below	306	57.6
	3001-5000	99	18.6
	5001-8000	61	11.5
	8001-12000	38	7.2
	Over 12000	27	5.1
Academic qualifications	High school and below	92	17.3
	Junior college	58	10.9
	Undergraduate	336	63.3
	Graduate	45	8.4
Total		531	100.0

#### 4.1.2. Non-response bias.

Non-response bias refers to the situation where individuals who do not respond to questionnaires may introduce bias into the research results [46]. This approach to addressing non-response follows the procedure proposed by Armstrong and Overton [47], who suggests that late respondents are more likely to resemble non-respondents than early respondents. This study addresses this issue by comparing the gender variables between early and late respondents. Gender was selected as the comparison variable because it is a stable demographic characteristic that is unlikely to be influenced by survey timing and provides a reliable indicator for detecting potential response bias. The 320 respondents who completed the survey in the early phase were considered as early respondents, while the 211 respondents who completed it in the later phase were deemed as late respondents. The chi-square test conducted between the early and late respondents reveals no significant difference in gender (p > 0.05). Therefore, the possibility of non-response bias is excluded.

### 4.2. Reliability and validity test

#### 4.2.1. Reliability analysis of scale data.

This study analyzes three constructs: sugar perception confusion, ambivalence attitude, and purchasing avoidance, with the latter further comprising delaying purchase and abandoning purchase [11]. To ensure measurement quality, five psychometric indicators were evaluated following established criteria. Factor loadings represent the correlation between observed items and their latent constructs, with a threshold greater than 0.6 being acceptable and ideally exceeding 0.7 [48–49]. Cronbach’s Alpha (α) measures internal consistency reliability among scale items and should exceed 0.7 [50]. The rho_ A coefficient is a reliability estimate unaffected by the number of items, with a recommended threshold above 0.7 [51–52]. Composite Reliability (CR) assesses the overall reliability of construct measurement and should be greater than 0.6 [49]. Average Variance Extracted (AVE) indicates the proportion of variance captured by a construct relative to measurement error, with values above 0.5 indicating good convergent validity [49].

As shown in Table 3, all measures exceeded their respective thresholds: factor loadings ranged from 0.748 to 0.941 (all > 0.7), Cronbach’s α ranged from 0.792 to 0.934 (all > 0.7), rho_ A ranged from 0.807 to 0.934 (all > 0.7), Composite Reliability ranged from 0.879 to 0.958 (all > 0.6), and AVE ranged from 0.710 to 0.883 (all > 0.5). These results confirm excellent reliability and convergent validity of the measurement model.

**Table 3:** Reliability and convergent validity of each construct.

Variables	Measurement Items	factor-loading	Cronbach’s Alpha	rho_A	Composite Reliability (CR)	Average Extraction Variance (AVE)
Sugar Perception Confusion	SPC1	0.748	0.792	0.807	0.879	0.710
	SPC2	0.896				
	SPC3	0.876				
Ambivalence Attitude	AA1	0.889	0.884	0.889	0.928	0.811
	AA2	0.930				
	AA3	0.882				
Delaying Purchase	DP1	0.940	0.934	0.934	0.958	0.883
	DP2	0.939				
	DP3	0.941				
Abandoning Purchase	AP1	0.926	0.920	0.921	0.949	0.862
	AP2	0.940				
	AP3	0.920				

Note: SPC = Sugar Perception Confusion; AA = Ambivalence Attitude; AP = Abandoning Purchase; DP = Delaying Purchase.

#### 4.2.2. Discriminant validity analysis.

Discriminant validity refers to how a set of items can effectively distinguish one variable from others. Two primary methods are employed to measure discriminant validity: the Fornell-Larcker criterion and the Heterotrait-Monotraitratio (HTMT). In the analysis using the Fornell-Larcker criterion, if the square root of the Average Variance Extracted (AVE) for a construct is significantly greater than its correlations with other constructs, and the indicators within that construct exhibit higher loadings on their corresponding construct, then that construct is considered to have discriminant validity. Higher values on the diagonal, representing the square roots of AVEs, indicate greater discriminant validity [53]. The results of the Fornell-Larcker criterion in Table 4 indicate that each construct (denoted by bold numbers) has an AVE square root greater than its correlations with other constructs, thus achieving discriminant validity (see Table 4). Furthermore, when HTMT values are below the recommended threshold of 0.90, discriminant validity does not pose a serious concern [54]. All values in Table 5 indicate that the HTMT ratios between constructs are below the critical value of 0.90. Therefore, the study results indicate that the discriminant validity is acceptable.

**Table 4:** Fornell–Larcker criterion.

	PA	SPC	AA
PA	***0.844***		
SPC	0.497	***0.842***	
AA	0.683	0.576	***0.901***

Note: SPC = Sugar Perception Confusion; AA = Ambivalence Attitude; PA = Purchasing Avoidance.

**Table 5:** Heterotrait–Monotrait Ratio (HTMT).

	PA	SPC	AA
PA			
SPC	0.576		
AA	0.744	0.681	

Note: SPC = Sugar Perception Confusion; AA = Ambivalence Attitude; PA = Purchasing Avoidance.

### 4.3. Path analysis

To evaluate the model structure, the study employs a bootstrapping procedure with 5000 resamples to obtain standardized β (β), t-values, and the coefficient of determination (R²) [52]. The model was utilized to estimate path coefficients and T-values. Path coefficients represent the strength and direction of the relationships between variables, revealing the interactions between observed and latent variables. On the other hand, R² values indicate the model’s explanatory power [40,55,56].

**Fig 2:**
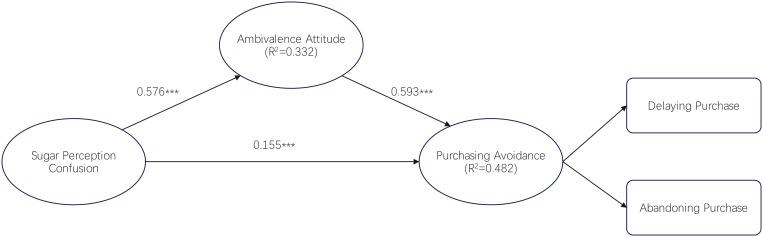
Standardized path coefficients and significance. Note:*p-value <0.05;**p-value<0.01;***p-value < 0.001;R² = coefficient of determination.

Table 6 and Fig 2 show that sugar perception confusion (β = 0.576, t = 16.102) and ambivalence attitude (β = 0.593, t = 14.571) positively impact purchasing avoidance. Thus, hypotheses H1 and H2 are supported. Sugar perception confusion (β = 0.155, t = 3.586) has a positive impact on ambivalence attitude, thus hypothesis H3 is supported.

**Table 6:** Results of model hypothesis testing.

Hypothesis	path	Standardized Path Coefficient	Sample Mean(M)	Standard Deviation (STDEV)	T Statistics	P Values	Result
H1	SPC - > PA	0.155	0.156	0.043	3.586***	0.000	Support
H2	AA - > PA	0.593	0.593	0.041	14.571***	0.000	Support
H3	SPC - > AA	0.576	0.576	0.036	16.102***	0.000	Support

Note: 1. SPC = Sugar Perception Confusion; AA = Ambivalence Attitude; PA = Purchasing Avoidance 2. *p-value < 0.05; ** p-value < 0.01; *** P-value < 0.001.

Based on Table 7, the mediation analysis reveals that ambivalence attitude significantly mediates the relationship between sugar perception confusion and purchasing avoidance (β = 0.342, p < 0.001). This indicates that sugar perception confusion not only directly influences purchasing avoidance (β = 0.155) but also operates through ambivalence attitude, which itself has a strong direct effect on purchasing avoidance (β = 0.593). The mediation effect suggests that when consumers encounter confusing sugar-related information, they develop conflicted attitudes toward sugar-sweetened beverages, which in turn amplifies their tendency to avoid purchases. The total effect of sugar perception confusion on purchasing avoidance is substantial (β = 0.497, combining direct and indirect effects), highlighting the critical role of clear communication in consumer decision-making.

**Table 7:** Mediating effect.

	Original Sample (O)	Sample Mean (M)	Standard Deviation (STDEV)	T Statistics (|O/STDEV|)	P Values	Conclusion
SPC - > AA - > PA	0.342	0.342	0.031	11.175	0.000	Presence of mediation

Note: SPC = Sugar Perception Confusion; AA = Ambivalence Attitude; PA = Purchasing Avoidance.

## 5. Discussion and limitation

Based on the information processing difficulty perspective, this research establishes and validates a structural equation model using PLS-SEM analysis to examine how information conflicts create consumer decision barriers. The findings reveal significant relationships with substantial effect sizes, providing theoretical insights and practical guidance for understanding passive avoidance mechanisms in health-related consumption contexts. The core empirical findings demonstrate that sugar perception confusion has a strong positive effect on ambivalent attitudes, indicating that information processing difficulties significantly increase consumer cognitive conflicts. Ambivalent attitudes show the strongest direct effect on purchasing avoidance, confirming that cognitive conflicts are the primary driver of avoidance behaviors. Additionally, sugar perception confusion has both significant direct effects and important indirect effects on purchasing avoidance through ambivalent attitudes. The mediation analysis reveals that ambivalent attitudes significantly mediate the relationship between sugar perception confusion and purchasing avoidance, indicating that confusion primarily leads to passive avoidance behaviors through cognitive burden rather than clear health cognitions. The theoretical contributions are manifested in three aspects: first, quantifying how information processing difficulties translate into cognitive conflicts, demonstrating substantial explanatory power for understanding ambivalent attitudes in health-related consumption contexts; second, the model exhibits strong predictive capability for purchasing avoidance behaviors; third, the significant mediation effect confirms that cognitive conflicts serve as the primary pathway through which information processing difficulties lead to behavioral avoidance, rather than through rational health-conscious decision-making.

### 5.1. Information processing optimization strategies for sugar perception confusion

Based on research findings, sugar perception confusion stems from specific information conflicts consumers face, including contradictions between health warnings and product marketing information, inconsistent information about sugar intake standards from different sources, and discrepancies between complex nutritional labels and simplified advertising claims. To mitigate this confusion, businesses should implement, businesses should implement systematic information clarification initiatives to reduce information processing barriers. First, standardize sugar content communication systems by adopting unified sugar content labeling methods, such as sugar content per 100 ml and percentage of daily recommended intake, providing visualized sugar content comparisons, and establishing simplified sugar content classification systems (low, medium, high sugar) to reduce information processing burden. Second, eliminate information conflict sources by providing clear core sugar content information on front-of-package to avoid consumers needing to interpret complex nutritional labels, coordinating marketing information with health information to eliminate contradictory statements, and providing quick sugar content understanding tools such as intuitive representations like “equivalent to X sugar cubes.” Third, simplify information architecture by developing decision support tools to help consumers process sugar information more effectively, including clear comparison frameworks showing sugar content relative to daily recommended intake, simplified nutritional labels with essential sugar information prominently displayed, and accessible explanations of sugar-related health implications without technical jargon.

### 5.2. Cognitive conflict management strategies for ambivalent attitudes

Given that ambivalent attitudes have the strongest effect on purchasing avoidance, businesses should prioritize strategies that reduce cognitive conflicts rather than simply providing more information. For multi-source information conflict management, businesses should monitor and respond to contradictory health information circulating in media and social networks, provide fact-based clarifications when conflicting sugar-related information emerges, and collaborate with health organizations to ensure message consistency. In supporting information processing, implement cognitive load reduction strategies including point-of-purchase decision aids that simplify sugar content evaluation, clear product categorization systems that reduce comparison complexity, and accessible customer education programs about reading and understanding sugar content information. Timely sales feedback and product review optimization are crucial for reducing cognitive conflicts. Businesses should promptly collect post-sales feedback, manage online evaluations, address negative comments swiftly, resolve consumer dissatisfaction, and prevent the spread of negative word-of-mouth. Additionally, cultivating key opinion leaders and spreading positive word-of-mouth is an important strategy for reducing uncertainty. Through influential opinion leaders using promotional articles, live streams, and advertisements to market products, when consumers are confused about various aspects of products, the positive word-of-mouth spread by these influential opinion leaders can help reduce consumer uncertainty.

### 5.3. Decision recovery strategies for purchasing avoidance

Based on the finding that delaying purchase represents temporary decision paralysis rather than permanent rejection, businesses can implement decision facilitation strategies to help consumers complete interrupted purchase decisions. For delaying purchase behaviors, businesses should provide immediate sugar content clarification at point-of-purchase, offer simplified choice architectures that reduce cognitive burden, and develop time-limited decision support to help consumers complete interrupted purchase decisions. Given that social influences significantly impact both delaying and abandoning purchases, businesses should ensure consistency of information across social channels, provide authoritative responses to sugar-related concerns in social media discussions, and facilitate peer-to-peer information sharing that reduces rather than increases confusion. The empirical results demonstrate that sugar perception confusion has a significant positive impact on delaying purchases, with confusion influenced by others having the most significant effect. From a comprehensive perspective, the influence of others can directly and significantly affect both delaying and abandoning purchase behaviors. To address this type of consumer confusion, businesses should take the specific measures mentioned above, then focus on consumers’ confusion regarding the consequences of increased sugar content, aiming to reduce contradictory attitudes and ultimately decrease the likelihood of delaying and abandoning purchase behaviors.

### 5.4. Research limitations

This study acknowledges several limitations. First, online survey methodology may not capture the full complexity of real-time information processing challenges consumers face during actual purchase decisions. Second, self-reported confusion measures may not fully reflect unconscious information processing difficulties or real-time decision-making challenges. Third, the cross-sectional design limits our understanding of temporal dynamics in confusion development. Regarding theoretical limitations, this study focused on information processing outcomes without examining specific sources and mechanisms of information conflicts that create these conflicts. Future research should investigate specific confusion influencing factors, including variations in media information quality across different channels, social network information transmission patterns that amplify or reduce confusion, educational background effects that moderate information processing capabilities, temporal dynamics of information exposure and confusion development, and individual difference factors that moderate information processing difficulties.

Important systemic considerations indicate that while this research provides business strategy insights, consumer information processing difficulties reflect broader systemic issues requiring multi-stakeholder coordination. The complete resolution of information conflicts requires coordinated efforts from regulatory bodies (for standardized labeling requirements), educational institutions (for health literacy development), media organizations (for accurate health communication), and healthcare providers (for consistent health guidance). Business interventions alone cannot address the complex information ecosystem that creates these processing difficulties. Future research should examine cross-stakeholder coordination mechanisms for improving information clarity, effectiveness of different information standardization approaches, and consumer information processing capacity building strategies that address both individual and systemic factors contributing to decision difficulties.

## References

[1] Walton J, Bell H, Re R, Nugent AP. Current perspectives on global sugar consumption: definitions, recommendations, population intakes, challenges and future direction. Nutr Res Rev. 2023;36(1):1–22. doi: 10.1017/S095442242100024X 34369326

[2] CDC. Sugar Sweetened Beverage Intake. 2022, Published 11 April 2022. Retrieved from https://www.cdc.gov/nutrition/data-statistics/sugar-sweetened-beverages-intake.html

[3] Singh GM, Micha R, Khatibzadeh S, Lim S, Ezzati M, Mozaffarian D, et al. Estimated Global, Regional, and National Disease Burdens Related to Sugar-Sweetened Beverage Consumption in 2010. Circulation. 2015;132(8):639–66. doi: 10.1161/CIRCULATIONAHA.114.010636 26124185 PMC4550496

[4] Pelletier JE, Graham DJ, Laska MN. Social norms and dietary behaviors among young adults. Am J Health Behav. 2014;38(1):144–52. doi: 10.5993/AJHB.38.1.15 24034689 PMC3876876

[5] Berens S, Funke J. A vignette study of option refusal and decision deferral as two forms of decision avoidance: Situational and personal predictors. PLoS One. 2020;15(10):e0241182. doi: 10.1371/journal.pone.0241182 33095825 PMC7584223

[6] Saranya P., Joji Alex N. Consumer Confusion on Cognitive Dissonance: A Conceptual Framework. NMIMS Management Review. 2024;32(1):14–20. doi: 10.1177/09711023241260519

[7] Boxall LR, Arden-Close E, James J, Appleton KM. Effects of dietary recommendations for reducing free sugar intakes, on free sugar intakes, dietary profiles and anthropometry: a randomised controlled trial. Br J Nutr. 2025;133(5):694–710. doi: 10.1017/S0007114525000339 39973355 PMC12055439

[8] Friedman MP. Consumer confusion in the selection of supermarket products. J Appl Psychol. 1966;50(6):529–34. doi: 10.1037/h0024048 5978050

[9] Bawden D, Holtham C, Courtney N. Perspectives on information overload. Aslib Proceedings. 1999;51(8):249–55. doi: 10.1108/eum0000000006984

[10] Jacoby J, Speller DE, Kohn CA. Brand Choice Behavior as a Function of Information Load. Journal of Marketing Research. 1974;11(1):63–9. doi: 10.1177/002224377401100106

[11] Walsh G, Mitchell V. The effect of consumer confusion proneness on word of mouth, trust, and customer satisfaction. European Journal of Marketing. 2010;44(6):838–59. doi: 10.1108/03090561011032739

[12] Festinger L. A Theory of Cognitive Dissonance. Stanford University Press. 1957. doi: 10.1515/9781503620766

[13] Leek S, Kun D. Consumer confusion in the Chinese personal computer market. Journal of Product & Brand Management. 2006;15(3):184–93. doi: 10.1108/10610420610668621

[14] Farley TA, Halper HS, Carlin AM, Emmerson KM, Foster KN, Fertig AR. Mass Media Campaign to Reduce Consumption of Sugar-Sweetened Beverages in a Rural Area of the United States. Am J Public Health. 2017;107(6):989–95. doi: 10.2105/AJPH.2017.303750 28426298 PMC5425869

[15] Morley BC, Niven PH, Dixon HG, Swanson MG, McAleese AB, Wakefield MA. Controlled cohort evaluation of the LiveLighter mass media campaign’s impact on adults’ reported consumption of sugar-sweetened beverages. BMJ Open. 2018;8(4):e019574. doi: 10.1136/bmjopen-2017-019574 29695387 PMC5922472

[16] Elliott-Green A, Hyseni L, Lloyd-Williams F, Bromley H, Capewell S. Sugar-sweetened beverages coverage in the British media: an analysis of public health advocacy versus pro-industry messaging. BMJ Open. 2016;6(7):e011295. doi: 10.1136/bmjopen-2016-011295 27436666 PMC4964256

[17] Scott WA. Brief Report : Measures Of Cognitive Structure. Multivariate Behav Res. 1966;1(3):391–5. doi: 10.1207/s15327906mbr0103_9 26825605

[18] Otnes C, Lowrey TM, Shrum LJ. Toward an Understanding of Consumer Ambivalence. J CONSUM RES. 1997;24(1):80–93. doi: 10.1086/209495

[19] Plambeck N, Weber K. CEO Ambivalence and Responses to Strategic Issues. Organization Science. 2009;20(6):993–1010. doi: 10.1287/orsc.1090.0471

[20] Conner M, Sparks P. Ambivalence and Attitudes. European Review of Social Psychology. 2002;12(1):37–70. doi: 10.1080/14792772143000012

[21] Prada M, Godinho CA, Garrido MV, Rodrigues DL, Coelho I, Lopes D. A qualitative study about college students’ attitudes, knowledge and perceptions regarding sugar intake. Appetite. 2021;159:105059. doi: 10.1016/j.appet.2020.105059 33271200

[22] Bogart LM, Elliott MN, Uyeda K, Hawes-Dawson J, Klein DJ, Schuster MA. Preliminary healthy eating outcomes of SNaX, a pilot community-based intervention for adolescents. J Adolesc Health. 2011;48(2):196–202. doi: 10.1016/j.jadohealth.2010.06.004 21257120 PMC3050639

[23] Thomas M, Nelson TF, Harwood E, Neumark-Sztainer D. Exploring parent perceptions of the food environment in youth sport. J Nutr Educ Behav. 2012;44(4):365–71. doi: 10.1016/j.jneb.2011.11.005 22507257

[24] Harris J, Frazier W III, Fleming-Milici F, Hubert P, Rodriguez-Arauz G, Grier S, et al. A qualitative assessment of US Black and Latino adolescents’ attitudes about targeted marketing of unhealthy food and beverages. Journal of Children and Media. 2019;13(3):295–316. doi: 10.1080/17482798.2019.1604394

[25] de Liver Y, van der Pligt J, Wigboldus D. Positive and negative associations underlying ambivalent attitudes. Journal of Experimental Social Psychology. 2007;43(2):319–26. doi: 10.1016/j.jesp.2006.02.012

[26] Beattie J, Baron J, Hershey JC, Spranca MD. Psychological determinants of decision attitude. Behavioral Decision Making. 1994;7(2):129–44. doi: 10.1002/bdm.3960070206

[27] Anderson CJ. The psychology of doing nothing: forms of decision avoidance result from reason and emotion. Psychol Bull. 2003;129(1):139–67. doi: 10.1037/0033-2909.129.1.139 12555797

[28] Lee H, Lalwani AK. Power Distance Belief and Consumer Purchase Avoidance: Exploring the Role of Cultural Factors in Retail Dynamics. Journal of Marketing Research. 2023;61(2):349–67. doi: 10.1177/00222437231182600

[29] White CM, Hafenbrädl S, Hoffrage U, Reisen N, Woike JK. Are groups more likely to defer choice than their members?. Judgm decis mak. 2011;6(3):239–51. doi: 10.1017/s1930297500001443

[30] Özkan E, Tolon M. The Effects of Information Overload on Consumer Confusion: An Examination on User Generated Content. boun. 2015;29(1):27–51. doi: 10.21773/boun.29.1.2

[31] Mitchell V, Papavassiliou V. Marketing causes and implications of consumer confusion. Journal of Product & Brand Management. 1999;8(4):319–42. doi: 10.1108/10610429910284300

[32] Wei Q, Lv D, Fu S, Zhu D, Zheng M, Chen S, et al. The Influence of Tourist Attraction Type on Product Price Perception and Neural Mechanism in Tourism Consumption: An ERP Study. Psychol Res Behav Manag. 2023;16:3787–803. doi: 10.2147/PRBM.S416821 37720172 PMC10504089

[33] Van Harreveld F, Nohlen HU, Schneider IK. The ABC of ambivalence: Affective, behavioral, and cognitive consequences of attitudinal conflict. Advances in Experimental Social Psychology. Elsevier. 2015. p. 285–324.

[34] Nai A. The Cadillac, the mother-in-law, and the ballot: Individual and contextual roots of ambivalence in Swiss direct democracy. Electoral Studies. 2014;33:292–306. doi: 10.1016/j.electstud.2013.06.010

[35] Pang J, Keh HT, Li X, Maheswaran D. “Every coin has two sides”: The effects of dialectical thinking and attitudinal ambivalence on psychological discomfort and consumer choice. J Consum Psychol. 2016;27(2):218–30. doi: 10.1016/j.jcps.2016.10.001

[36] Krejcie RV, Morgan DW. Determining Sample Size for Research Activities. Educational and Psychological Measurement. 1970;30(3):607–10. doi: 10.1177/001316447003000308

[37] Yao Y, Wang P, Jiang Y, Li Q, Li Y. Innovative online learning strategies for the successful construction of student self-awareness during the COVID-19 pandemic: Merging TAM with TPB. Journal of Innovation & Knowledge. 2022;7(4):100252. doi: 10.1016/j.jik.2022.100252

[38] Ermeç Sertoğlu A, Kavak B. A More Comprehensive View of Consumer Confusion: Scale Development. Journal of International Consumer Marketing. 2017;29(4):265–76. doi: 10.1080/08961530.2017.1295297

[39] Priester JR, Petty RE. The gradual threshold model of ambivalence: relating the positive and negative bases of attitudes to subjective ambivalence. J Pers Soc Psychol. 1996;71(3):431–49. doi: 10.1037//0022-3514.71.3.431 8831157

[40] Hair JF, Risher JJ, Sarstedt M, Ringle CM. When to use and how to report the results of PLS-SEM. EBR. 2019;31(1):2–24. doi: 10.1108/ebr-11-2018-0203

[41] Gefen D, Rigdon EE, Straub D. Editor’s Comments. MIS Quarterly. 2011;35(2):iii-A7. doi: 10.2307/23044042

[42] Khan GF, Sarstedt M, Shiau W-L, Hair JF, Ringle CM, Fritze MP. Methodological research on partial least squares structural equation modeling (PLS-SEM). INTR. 2019;29(3):407–29. doi: 10.1108/intr-12-2017-0509

[43] Petter S, Straub D, Rai A. Specifying Formative Constructs in Information Systems Research1. MIS Quarterly. 2007;31(4):623–56. doi: 10.2307/25148814

[44] Anderson JC, Gerbing DW. Structural equation modeling in practice: A review and recommended two-step approach. Psychological Bulletin. 1988;103(3):411–23. doi: 10.1037/0033-2909.103.3.411

[45] Hulland J. Use of partial least squares (PLS) in strategic management research: a review of four recent studies. Strat Mgmt J. 1999;20(2):195–204. doi: 10.1002/(sici)1097-0266(199902)20:2<195::aid-smj13>3.0.co;2-7

[46] Shiau W-L, Yuan Y, Pu X, Ray S, Chen CC. Understanding fintech continuance: perspectives from self-efficacy and ECT-IS theories. IMDS. 2020;120(9):1659–89. doi: 10.1108/imds-02-2020-0069

[47] Armstrong JS, Overton TS. Estimating Nonresponse Bias in Mail Surveys. Journal of Marketing Research. 1977;14(3):396–402. doi: 10.1177/002224377701400320

[48] Hair JF, Hult GTM, Ringle CM, Sarstedt M. A primer on partial least squares structural equation modeling (PLS-SEM). 2 ed. Sage Publications. 2017.

[49] Fornell C, Larcker DF. Evaluating Structural Equation Models with Unobservable Variables and Measurement Error. Journal of Marketing Research. 1981;18(1):39–50. doi: 10.1177/002224378101800104

[50] Nunnally JC, Bernstein IH. Psychometric theory. 3rd ed. McGraw-Hill. 1994.

[51] Dijkstra TK, Henseler J. Consistent Partial Least Squares Path Modeling1. MIS Quarterly. 2015;39(2):297–316. doi: 10.25300/misq/2015/39.2.02

[52] Hair JF, Hult GT, Ringle C, Sarstedt M. A Primer on Partial Least Squares Structural Equation Modeling (PLS-SEM)-Joseph F. Hair, Jr., G. Tomas M. Hult, Christian Ringle, Marko Sarstedt.

[53] Hair JF, Sarstedt M, Ringle CM, Mena JA. An assessment of the use of partial least squares structural equation modeling in marketing research. J of the Acad Mark Sci. 2011;40(3):414–33. doi: 10.1007/s11747-011-0261-6

[54] F. Hair Jr J, Sarstedt M, Hopkins L, G. Kuppelwieser V. Partial least squares structural equation modeling (PLS-SEM). European Business Review. 2014;26(2):106–21. doi: 10.1108/ebr-10-2013-0128

[55] Hair J, Hair Jr JF, Sarstedt M, Ringle CM, Gudergan SP. Advanced issues in partial least squares structural equation modeling. SAGE Publications. 2023.

[56] Shiau W-L, Luo MM. Factors affecting online group buying intention and satisfaction: A social exchange theory perspective. Computers in Human Behavior. 2012;28(6):2431–44. doi: 10.1016/j.chb.2012.07.030

